# Multi-omics integrated analysis to explore the molecular mechanisms of Xinkai Kujiang formula in treating gastric intestinal metaplasia in rats

**DOI:** 10.3389/fphar.2026.1881703

**Published:** 2026-08-26

**Authors:** Jiao Xiang, Yuqin Cheng, Zhaoling Wang, Jing Han, Ling Xiao, Jianping Wu, Yufen Meng, Zhaolai Hua, Bin Lu, Chun Cheng, Junfeng Zhang

**Affiliations:** 1 School of Medicine, Nanjing University of Chinese Medicine, Nanjing, Jiangsu, China; 2 Laboratory Animal Center, Nanjing University of Chinese Medicine, Nanjing, Jiangsu, China; 3 Institute of Tumour Prevention and Control, Yangzhong People’s Hospital, Zhenjiang, Jiangsu, China; 4 Department of Oncology, Yangzhong People’s Hospital, Zhenjiang, Jiangsu, China

**Keywords:** gastric intestinal metaplasia, gut microbiota, scRNA-sequencing, transcriptome, Xinkai Kujiang formula

## Abstract

**Background:**

Gastric intestinal metaplasia (GIM) is a typical precancerous lesion of gastric cancer (PLGC). Previous studies have demonstrated that Xinkai Kujiang formula can effectively alleviate GIM, but its underlying mechanism remains largely unclear.

**Methods:**

The GIM rat model was established using 2% sodium salicylate and 20 mmol/L sodium deoxycholate, and then the rats were treated with Banxia Xiexin Decoction (BXD) and Xinkai Kujiang Decoction (XKD) for 4 weeks. Multi-omics analyses including 16 S ribosomal RNA gene sequencing, transcriptomics, single-cell RNA sequencing, network pharmacology, and component identification were performed to explore the therapeutic mechanisms of Xinkai Kujiang formula on GIM.

**Results:**

In the model rats, severe gastric mucosal atrophy was observed, characterized by disordered glands and goblet cells. Following intervention with BXD and XKD, gastric mucosal thickness was restored, glandular structures became regularly arranged, and the number of metaplastic goblet cells markedly decreased. Microbiota profiling of gastric mucosa revealed significant enrichment of *Lactobacillus* and *Enterococcus* in the model group. These abundances were reduced in the BXD group, and short-chain fatty acid-producing bacteria such as *Alistipes* and *Lachnospira* were enriched. In the intestine, opportunistic pathogens like *Streptococcus* and *Enterococcus* were enriched in the model group, whereas *Corynebacterium* and *Bifidobacterium* were enriched in the XKD group. Transcriptomic analysis presented that BXD upregulated innate immune-related genes in the gastric mucosa, and single-cell RNA sequencing (scRNA-Seq) showed that XKD alleviated GIM by inhibiting the VEGF and HIF-1α pathways, reducing angiogenesis, suppressing inflammatory infiltration, and regulating energy metabolism.

**Conclusion:**

BXD and XKD improve gastrointestinal microbiota disorders and metabolic disorders, enhance gastric mucosal immunity, and inhibit the VEGF and HIF-1α pathway. Collectively, these multi-omics data provide novel insights into the therapeutic mechanisms of Xinkai Kujiang formula for GIM.

## Graphical Abstract

**Figure:**
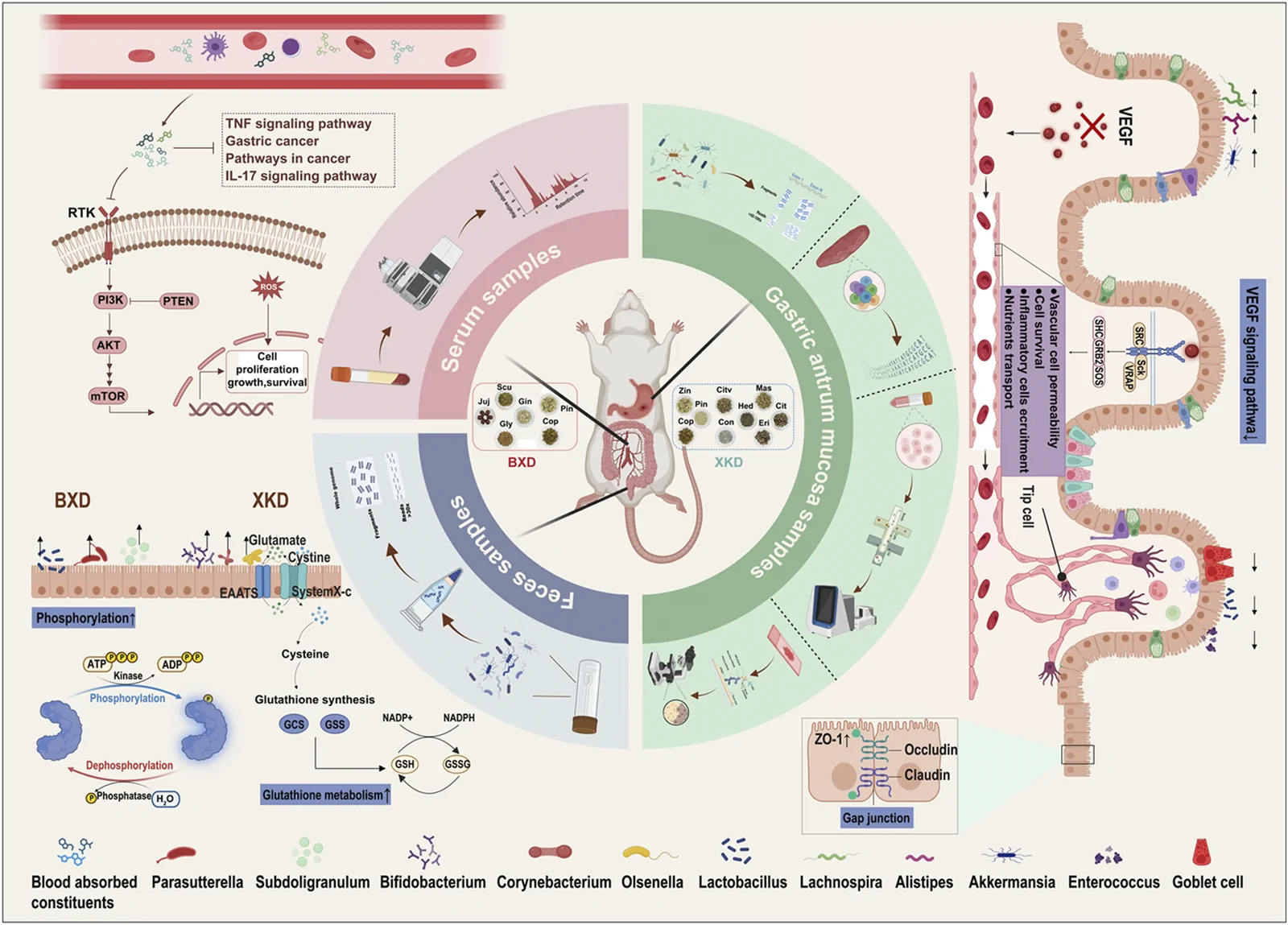

## 1 Introduction

Gastric cancer (GC) is one of the most common malignant tumors. Correa’s cascade hypothesis holds that normal gastric mucosa undergoes chronic gastritis, atrophic gastritis, intestinal metaplasia, and dysplasia before ultimately developing into GC. Gastric intestinal metaplasia (GIM) is the most important stage in the development of GC, characterized by the replacement of normal gastric mucosa with intestinal-type epithelium, representing a typical precancerous lesion of gastric cancer (PLGC) (He et al., 2022). As early as 1999, You et al. (1999) found that GIM increased the risk of GC by 17.4–29.3-fold in patients with chronic gastritis. Additionally, Dhingra et al. (2020) conducted a case-control study including 358 GIM patients and found that the annual incidence rate of gastric adenocarcinoma in the GIM group (0.8/1,000 person-years) was significantly higher than that in the control group (0.07/1,000 person-years). Therefore, the prevention and treatment of GIM is of great significance in reducing the incidence of GC.

In 2022, China officially issued the Chinese Integrated Guideline on Clinical Management of Gastric Precancerous Conditions and Lesions for the first time, which stated that dysplasia (also known as intraepithelial neoplasia) is a definite PLGC, while atrophic gastritis, GIM, and dysplasia are broadly classified as PLGC (Wang et al., 2022). Accumulating evidence from Traditional Chinese Medicine (TCM) research has proved that Xinkai Kujiang formula is a mainstay treatment for PLGC, with Banxia Xiexin Decoction (BXD) serving as one of its classic formulas (Li et al., 2021a). Numerous studies have demonstrated that BXD can effectively improve pathological changes such as gastric mucosal atrophy and intestinal metaplasia in patients with PLGC and has been recognized as the preferred prescription for syndrome-based treatment of PLGC (Li et al., 2022). Our previous study demonstrated that Xinkai Kujiang Decoction (XKD) significantly reduced the degree of esophageal dysplasia and GIM in patients, however, the detailed mechanisms remain largely unclear (Zhou et al., 2018).

Numerous studies have shown that gut microbiota dysbiosis is closely associated with PLGC, and certain bacteria can even induce GC. For example, inoculation of antral tissues, biopsy samples, or gastric juice from patients with chronic superficial gastritis, GIM, or GC has been shown to successfully induce PLGC in mice (Kwon et al., 2022). In *Helicobacter pylori* (Hp)-negative patients, Park et al. (2019) found that the relative abundances of *Actinobacteria* and *Proteobacteria* gradually increased during GC progression, whereas that of *Cyanobacteria* decreased. Fu et al. (2024) confirmed that the surface protein TMPC (membrane-associated lipoprotein of *T. pallidum*) of *Streptococcus anginosus* interacts with the annexin A2 (ANXA2) receptor on gastric epithelial cells, activating the mitogen-activated protein kinase (MAPK) signaling pathway and promoting gastric carcinogenesis. Our previous study established a rat model of GIM by simulating bile reflux with deoxycholic acid and found significant alterations in the gastric microbiota. The relative abundances of *Actinobacteria* and *Proteobacteria* were markedly increased, while the abundance of *Fusobacteria* was significantly reduced. Moreover, *Limosilactobacillus* and *Ligilactobacillus* were significantly enriched in the GIM group (Xu et al., 2023). Therefore, the gastric microbiota represents a potential target for the prevention and treatment of GIM.

With the development of single-cell RNA sequencing (scRNA-seq), the technique provided new perspective for elucidating the cellular and molecular mechanisms underlying gastric mucosal lesions. For example, Zhang et al. (2019) found that a key mechanism in the development of GIM and GC may be associated with the acquisition of an intestinal stem cell-like phenotype by neck mucous cells. Kim et al. (2022) performed scRNA-seq on tumor tissues from patients with advanced GC before and after chemotherapy, and found that chemotherapy could increase natural killer (NK) cell infiltration, promote macrophage repolarization, and enhance antigen presentation functions in tumor tissues. Li et al. (2024) comparatively analyzed the cellular atlas of three key pathological stages during GC progression (gastritis, GIM, and GC), finding that with the progression of gastric mucosal lesions, the proportion of epithelial cells gradually decreases while the proportion of myeloid cells gradually increases. Therefore, the application of scRNA-seq is of great value for revealing the underlying mechanisms of TCM prescriptions in the treatment of GIM.

Therefore, this study aimed to conduct an integrated multi-omics correlation analysis of gastrointestinal microbiota, serum metabolites, gastric mucosal genes, and scRNA-seq data to initially explore the potential mechanisms of the Xinkai Kujiang formula in GIM rats. This integrated multi-omics provides a more precise theoretical basis for the clinical application of Xinkai Kujiang formula, and offers novel perspectives for the prevention and treatment of GC.

## 2 Materials and methods

### 2.1 Preparation of BXD and XKD

BXD comprises *Pinelliae Rhizoma Praeparatum* (Pin, 12 g), *Scutellariae Radix* (Scu, 9 g), *Zingiberis Rhizoma* (Zin, 9 g), *Ginseng Radix et Rhizoma* (Gin, 9 g), *Glycyrrhizae Radix et Rhizoma Praeparata Cum Melle* (Gly, 9 g), *Coptidis Rhizoma* (Cop, 3 g), *Jujubae Fructus* (Juj, 6 g). XKD comprises *Pinelliae Rhizoma Praeparatum* (Pin, 10 g), *Zingiberis Rhizoma* (Zin, 6 g), *Coptidis Rhizoma* (Cop, 3 g), *Hedyotis diffusa Willd* (Hed, 20 g), *Erinaceus europaeus Linnaeus* (Eri, 6 g), *Concha Arcae* (Con, 20 g), *Citri reticulatae Pericarpium Viride* (CitV, 6 g), *C. reticulatae Pericarpium* (Cit, 6 g), *Massa Medicata Fermentata* (Mas, 10 g). All medicinal materials were purchased from Beijing Tongrentang Pharmacy (Nanjing, China). The dosage (crude drug content) of rats intragastric administration was calculated based on the body surface area ratio: 5.13 g/kg for BXD and 7.83 g/kg for XKD. Deoxycholate sodium (Beyotime Biotech. Inc., ST 2049, Shanghai, China) was prepared as a 20 mmol/L deoxycholate sodium solution. Sodium salicylate (Sigma-Aldrich, S3007, United States of America) was prepared as 2% sodium salicylate solution and stored at 4 °C.

### 2.2 Preparation of experimental animals

Male specific pathogen-free (SPF) Sprague-Dawley (SD) rats, 5–6 weeks old, were obtained from Zhejiang Weitong Lihua Experimental Animal Technology Co., Ltd (license No. SCXK (Zhejiang) 2021–0011). A total of 24 rats were kept at the Animal Center of Nanjing University of Chinese Medicine, maintained on a 12 h light-dark cycle, with temperature at 23 °C–25 °C and relative humidity of 50%–70%.

### 2.3 Treatment of BXD and XKD in GIM rats

Following a 7-day adaptive feeding period, male SD rats were randomly divided into control and model groups. The modeling procedure was established according to the previous literature (Xu et al., 2023), summarized as follows: rats in the model group were treated daily with 2 mL cold solution of 2% sodium salicylate and had free access to drinking water containing 20 mmol/L sodium deoxycholate, with standard diet. After 12 weeks, the presence of glandular atrophy and reduction in antral mucosa thickness, along with an increase in goblet cells, was considered indicative of successful establishment of the GIM model. The GIM rats were then randomly assigned to three groups: model group, BXD group, and XKD group (n = 6 per group). Dosage selection was determined based on our preliminary experimental findings (Zhou et al., 2018). Overall, the control and model groups received sterilized water by gavage, the BXD group received 1.5 g/mL BXD decoction, and the XKD group received 2.2 g/mL XKD decoction. This intervention was administered continuously for 4 weeks, with a gavage volume of 1.7 mL/500 g body weight.

### 2.4 Blood-entering components and network pharmacology analysis of BXD and XKD

For serum metabolomics analysis, six rats were included in each group. Before the experiment, rats were fasted for 12 h but had free access to water. Rats in the BXD and XKD groups were administered drugs first; two hours later, all rats were anesthetized with isoflurane, and whole blood was collected from the abdominal aorta, then left to stand, centrifuged, and processed to obtain serum. Subsequent experimental procedures were carried out according to previously reported methods (Xu et al., 2023). Firstly, 400μL serum aliquot was mixed with 40 μL of 2 mol/L hydrochloric acid, vortexed for 1 min, and incubated at 4 °C for 15 min. This vortex-incubation cycle was repeated four times. Next, 1.6 mL of acetonitrile was added, followed by vertexing for 5 min and centrifugation at 12,000 rpm and 4 °C (RCF = 13,800 × g, R = 8.6 cm) for 5 min. The supernatant (1.8 mL) was transferred to a new tube and evaporated under nitrogen. The dried residue was redissolved in 150 μL of 80% methanol containing 10 μg/mL internal standard, vortexed for 5 min, and then centrifuged again at 12,000 rpm (RCF = 13,800 × g, R = 8.6 cm) at 4 °C for 5 min. From each sample, 20 μL was taken and pooled to prepare quality control (QC) samples. LC-MS/MS analysis was carried out on a Vanquish UHPLC system (Thermo Fisher Scientific) fitted with a Waters UPLC BEH C18 column (1.7 μm, 2.1 × 100 mm), with an injection volume of 5 μL, and the flow rate was maintained at 0.5 mL/min. The mobile phase comprised 0.1% formic acid in water (A) and 0.1% formic acid in acetonitrile (B). The Q Exactive Focus mass spectrometer coupled with Xcalibur software was used to acquire MS and MS/MS data in IDA acquisition mode. We performed the experiment following the compound identification methods from the referenced study (Wang et al., 2025). Specifically, the raw MS data were imported into XCMS software for retention time correction, peak detection, peak extraction, peak integration, and peak alignment. Then, peaks were identified based on MS/MS data by matching against an in-house secondary mass spectrometry database along with the corresponding fragmentation patterns.

Targets of the blood-entering components were retrieved from the TCMSP database (http://tcmspw.com/tcmsp.php), using the criteria of oral bioavailability (OB) ≥ 30% and drug-likeness (DL) ≥ 0.18 (Wu et al., 2025). The UniProt human gene Excel table was downloaded for target-gene conversion. Relevant genes were retrieved by querying the GeneCards (https://www.genecards.org/) using the keyword “gastric intestinal metaplasia”, with genes having a Relevance score ≥10 being retained, and from the OMIM (https://omim.org/) using the same keyword, followed by merging and deduplication. The converted TCM targets and disease-related genes were intersected to identify common targets. Kyoto Encyclopedia of Genes and Genomes (KEGG) signaling pathway enrichment analysis of the intersecting genes was carried out using the ClusterProfiler package in R software (version 4.4.0), with screening criteria defined as *P* value cutoff = 0.05 and *Q* value cutoff = 0.05.

### 2.5 Gastric mucosa and gut microbiota sequencing

Total DNA was extracted from gastric antrum and fecal samples using the E. Z.N.A. DNA Kit (Omega Bio-tek, Norcross, GA, United States of America). The V3-V4 region of the bacterial 16 S rRNA gene was amplified via polymerase chain reaction (PCR) with the primers 341 F (5′-CCTAYGGGRBGCASCAG-3′) and 806 R (5′-GGACTACNNGGGTATCTAAT-3′). PCR products were extracted from a 2% agarose gel and further purified using the AxyPrep DNA Gel Extraction Kit (Axygen Biosciences, Union City, CA, United States of America). The products were quantified using Qubit 3.0 fluorometer (Thermo Fisher Scientific, Waltham, MA, United States of America). Illumina sequencing libraries were prepared using the Nextflex Rapid DNA-Seq Kit (Bioo Scientific, Austin, TX, United States of America). All samples were processed in a single batch for DNA extraction, library construction, and sequencing to minimize batch effects. Six biological replicates were included for each group. Subsequently, the amplicon library underwent paired-end sequencing (2 × 250) on an Illumina MiSeq platform (Shanghai BIOZERON Co., Ltd.) in accordance with established procedures (Luo et al., 2020).

The sequences were quality-checked and clustered into *de novo* operational taxonomic units (OTUs) at a 97% similarity threshold using UPARSE software (version 7.0.1001). The alpha-diversity (Chao1, ACE, Shannon, and Simpson) was computed using Mothur (version 1.21.1), and the beta-diversity was evaluated using UniFrac. Principal component analysis (PCA) was performed using the vegan package in R to compare microbial community structures, and the results were visualized using Origin 2022 to illustrate community composition and clustering. To identify potential bacterial taxa among groups, the linear discriminant analysis (LDA) effect size (LEfSe) analysis was employed, which combines linear discriminant analysis with the Kruskal–Wallis non-parametric test. Features with an LDA score >2.0 and *P* < 0.05 were considered significantly enriched. Microbial functions were predicted using Phylogenetic Investigation of Communities by Reconstruction of Unobserved States (PICRUSt) based on the Kyoto Encyclopedia of Genes and Genomes (KEGG) database (*P* value Cutoff = 0.05, *Q* value Cutoff = 0.05). Visualization of the LEfSe results and PICRUSt-predicted functional profiles was conducted using the online platform Xiantao Academic (https://www.xiantaozi.com/).

### 2.6 Transcriptome analysis of gastric antral mucosa

Total RNA was extracted from gastric antral tissues using TRIzol reagent (Invitrogen™, Thermo Fisher Scientific, United States of America) and assessed for quality and concentration using a Nanodrop ND-2000 spectrophotometer (NanoDrop Technologies, United States of America). Only RNA samples meeting the following criteria were considered high-quality and used for library preparation: OD260/280 = 1.8–2.2, OD260/230 ≥ 2.0, RIN ≥6.5, 28 S:18 S ≥ 1.0, and yield >1 μg. All RNA samples were processed in a single batch for library construction and sequencing to minimize batch effects. Six biological replicates were included for each group. Paired-end libraries (150 bp × 2) were sequenced on the Illumina NovaSeq 6,000 platform (Shanghai BIOZERON Co., Ltd.). Gene expression levels were calculated by counting clean reads aligned to the genomic region. The expression level of each gene/transcript was then quantified as FPKM (fragments per kilobase of exon model per million mapped fragments) based on the alignment results of all samples to the reference genome. Differentially expressed genes (DEGs) between the two groups were identified with thresholds of *P* < 0.05, *FDR* ≤ 0.05 and *|*log*2 fold change (FC)|* ≥ 1. Subsequently, KEGG pathway enrichment analysis of the DEGs was carried out using the clusterProfiler package in R to identify the associated signaling pathways.

### 2.7 Untargeted serum metabolomics analysis

Metabolites were extracted from serum in accordance with the procedures reported previously (Liu et al., 2024). A 100μL serum sample was mixed with 400 μL of extraction solvent (MeOH:ACN = 1:1 v/v), vortexed for 30 s, then sonicated in a 4 °C water bath for 10 min and kept at −40 °C for 1 h to precipitate proteins. After centrifugation at 12,000 rpm (RCF = 13,800 × g, R = 8.6 cm) for 15 min at 4 °C, the supernatant was collected into a clean glass vial for LC-MS/MS analysis, and each group included six replicates.

The LC-MS/MS system consisted of a Vanquish UHPLC coupled to an Orbitrap Exploris 120 mass spectrometer (Thermo Fisher Scientific), equipped with a Waters ACQUITY UPLC BEH Amide column (2.1 mm × 50 mm, 1.7 μm). Mobile phases were 25 mmol/L ammonium acetate with 25 mmol/L ammonium hydroxide (pH 9.75) as phase A and acetonitrile as phase B. The autosampler was kept at 4 °C, and the injection volume was 2 μL. MS/MS data were acquired in Information Dependent Acquisition (IDA) mode using Xcalibur software. The ESI source settings were: sheath gas 50 Arb, auxiliary gas 15 Arb, capillary temperature 320 °C, full MS resolution 60,000, MS/MS resolution 15,000, collision energy SNCE 20/30/40, and spray voltage ±3.8 kV (positive) or −3.4 kV (negative) (Zhao et al., 2025).

Raw data were converted to mzXML format via ProteoWizard (version 3.0.8789) and then processed with an R package (based on XCMS) for peak detection, extraction, alignment, and integration. Metabolites were identified using R and BiotreeDB (version 2.1) with a cutoff of 0.3. Data were log-transformed and mean-centered (CTR) in SIMCA (version 16.0.2) for principal component analysis (PCA). Differential metabolites between groups were selected using *P* < 0.05 and VIP ≥1.0. KEGG pathway enrichment of these metabolites was performed with the clusterProfiler R package. Figures were prepared using the Xiantao Academic online platform (https://www.xiantaozi.com/).

### 2.8 Single-cell transcriptomic analysis of gastric mucosa

Gastric antrum tissues were collected from three rats per group and minced on ice into approximately 1 mm^3^ pieces. Tissue dissociation was performed using Tissue Dissociation Kit (Miltenyi Biotec, Bergisch Gladbach, Germany) at 37 °C for 30 min. After dissociation, the single-cell suspension was centrifuged for 5 min at 500 × g at 4 °C.

After the supernatant was removed, the cells were resuspended in the red blood cell lysis buffer (Miltenyi Biotec, 130–094-183, Bergisch Gladbach, Germany) to lyse red blood cells and washed with PBS containing 0.04% BSA (Sigma, B2064, United States of America). The suspension was filtered through a 35 μm cell strainer (Corning Falcon, 352,235). Finally, cells were stained with Draq7 (Abcam, ab109202) and Calcein AM (Thermo Fisher Scientific, C1430) for cell viability assessment. Single-cell capture and library construction were performed using the BD Rhapsody Single-Cell Analysis System (BD Biosciences, Franklin Lakes, NJ). After library preparation, the libraries were quantified using the Agilent 2,200 Bioanalyzer (Agilent Technologies, Palo Alto, CA) and the Qubit 4.0 (Thermo Fisher Scientific, United States of America). Finally, the libraries were sequenced on the Illumina NovaSeq 6,000 (San Diego, CA), and 300 bp reads (including 150 bp paired-end reads) were generated (Xiang et al., 2025).

Downstream clustering analysis and visualization were performed using the R package Seurat (version 4.4.0). Low-quality cells were filtered with the following criteria: (i) > 5,000 or <200 genes detected; (ii) mitochondrial gene percentage >10%. Following quality control, the gene expression matrix was normalized using the NormalizeData function, followed by principal component analysis (PCA) based on the top 2,000 highly variable genes. Clustering analysis was performed using the built-in clustering algorithm of the Seurat package. The t-distributed stochastic neighbor embedding (t-SNE) dimensionality reduction analysis identified 14 distinct cell clusters. Cell cycle stages were assigned with the CellCycleScoring function in Seurat. Differentially expressed genes (DEGs) in gastric antrum mucosal tissues were analyzed between the control group, BXD group, XKD group, and the model group using the FindMarkers function. Genes with *P* ≤ 0.05 and *|*log*2 fold change (FC)|* ≥ 0.25 were defined as significant DEGs, which were visualized via volcano plots. Gene ID conversion was performed using the ‘org.Hs.e.g.,.db’ package, followed by Kyoto Encyclopedia of Genes and Genomes (KEGG) enrichment analysis performed with the enrichKEGG and gseKEGG functions. Intercellular communication analysis was conducted using CellChat (version 3.16.0). The ligand-receptor pattern was used to demonstrate the number and intensity of interactions between clusters. Expression patterns of vascular endothelial growth factor (VEGF) ligand-receptor pairs across different cell types in the four experimental groups were visualized with the netVisual_bubble function. A heatmap of the expression levels of genes associated with the VEGF signaling pathway was generated using the pheatmap package (version 1.0.12).

### 2.9 Gastric mucosal tissue staining

Gastric antrum tissue (n = 6 per group) was fixed in 4% paraformaldehyde (Servicebio, G1101-15ML), then embedded in paraffin and cut into 4-μm-thick sections. The sections were deparaffinized with xylene (Sinopharm Chemical Reagent Co., Ltd., 10023418) for 10 min, then rehydrated through a graded ethanol series (100%, 95%, 85%, 75%) for 5 min each, followed by a 5-min soak in distilled water. Hematoxylin and eosin (H&E) staining was then performed using H&E staining solution (Servicebio, G1005) according to the following protocol: sections were stained with hematoxylin for 3–5 min, rinsed in running water, treated with bluing reagent, and washed again; then stained with eosin for 5 min, and mounted with neutral gum. Alcian blue-periodic acid-Schiff (AB-PAS) staining was carried out using an AB-PAS staining kit (Servicebio, G1049). Sections were stained with Alcian blue for 5 min and washed in running water for 2 min, oxidized in periodic acid for 15 min, rinsed twice in distilled water, incubated in Schiff reagent for 30 min in the dark, and washed again in running water. Counterstaining was performed with hematoxylin for 3–5 min, followed by bluing, dehydration, clearing, and mounting were implemented as described above.

Immunohistochemical (IHC) staining was conducted for gastric mucosal tissues. After deparaffinization and rehydration, the sections were boiled in antigen retrieval buffers (Servicebio, G1202, pH 6.0) and immersed with 3% hydrogen peroxide to block endogenous peroxidase activity. The tissues were blocked with 3% bovine serum albumin (BSA) for 30 min, followed by overnight incubation at 4 °C with the following primary polyclonal antibodies: anti-Bves (Invitrogen Corporation; PA5-102304,1:4,000 dilution), anti-Dmbt1 (Abcam; ab316185, 1:10,000 dilution), anti-ZO1 (Proteintech; 21773-1-AP, 1:10,000 dilution), anti-ZO1 (Thermo Fisher Scientific; 11–4,204-82, 1:10,000 dilution), anti-MUC2 (Servicebio; GB11344, 1:4,000 dilution), anti-MUC6 (Servicebio; GB15114, 1:4,000 dilution), anti-PGC (Proteintech; 28532-1-AP, 1:10,000 dilution), anti-VEGF (Servicebio; GB15165, 1:400 dilution), anti-HIF-1α (Servicebio; GB151339, 1:800 dilution), anti-CD31 (Servicebio; GB120008, 1:800 dilution). The sections were then warmed to room temperature, washed three times with PBS, and incubated with appropriate secondary antibodies at 37 °C for 50 min. Finally, sections were stained with diaminobenzidine (Servicebio, G1212-200 T), counterstained with hematoxylin, dehydrated, mounted, and observed under a microscope. All stained sections were quantified using ImageJ software, with results expressed as the percentage of positive area (Area%).

### 2.10 Multi-omics correlation network construction

Spearman correlation analysis was performed between LDA-identified gastric genera and gastric mucosal DEGs in the BXD and XKD groups. Correlations with |r| > 0.7 and *P* < 0.05 were considered significant. Additionally, correlations between LDA-screened intestinal genera and serum differential metabolites, as well as between serum metabolites and gastric mucosal DEGs, were analyzed to construct an intestinal genera-serum metabolites-gastric mucosal DEGs association network. All analyses were performed using R software.

### 2.11 Statistical analysis

Statistical analysis was conducted using SPSS 26.0. For data that followed a normal distribution and had homogeneous variance between two groups, an unpaired Student’s *t-*test was used. Comparisons among multiple groups were evaluated by one-way ANOVA, followed by Tukey’s *post hoc* test. *P*-value less than 0.05 was considered statistically significant.

## 3 Results

### 3.1 Analysis of blood-entry components of Xinkai Kujiang formula

**FIGURE 1:**
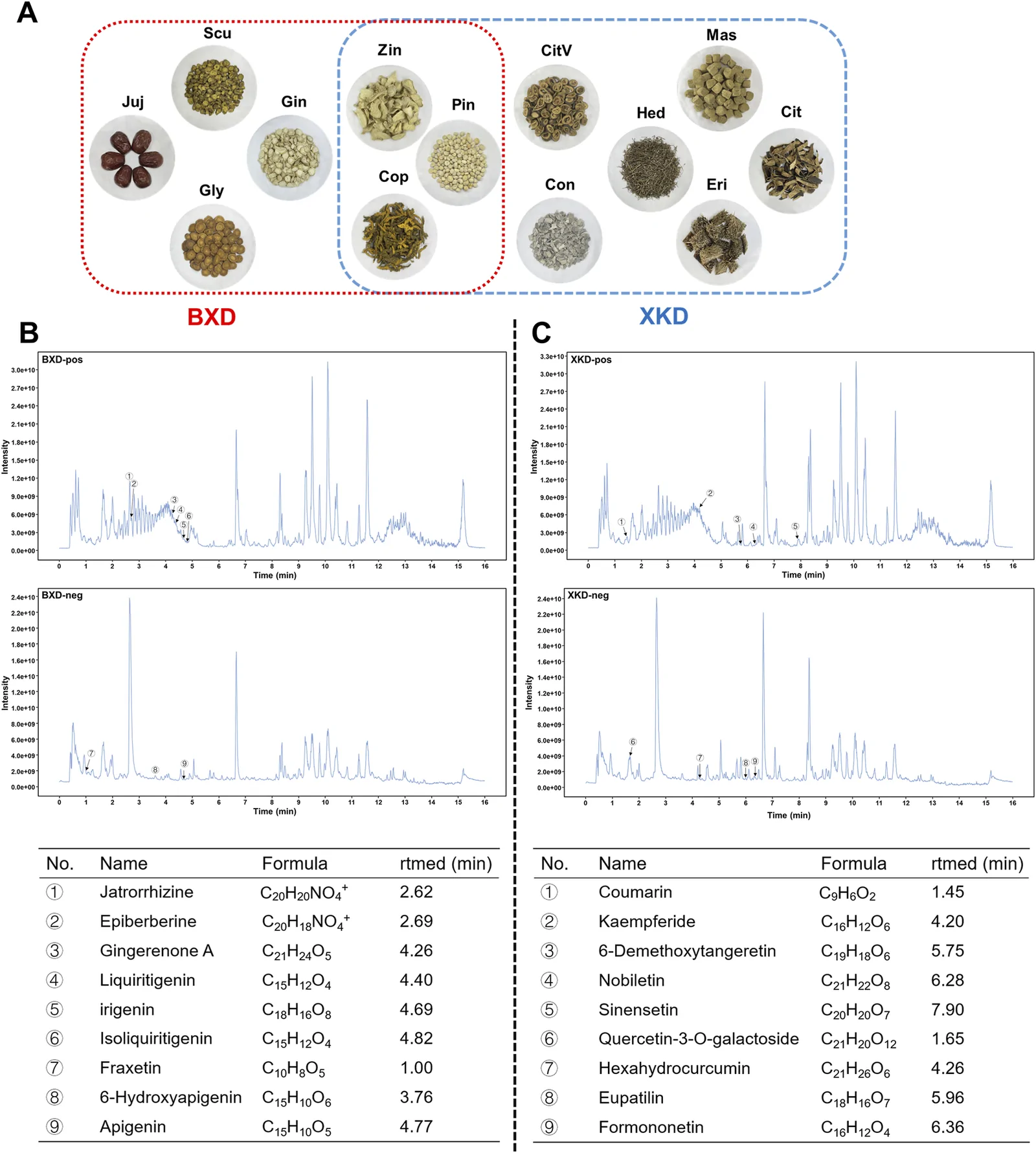
Analysis of blood-entry components of Xinkai Kujiang formula. **(A)** BXD and XKD comprise seven and nine Chinese herbal medicines, respectively. The both formulas shared *Pinelliae rhizoma praeparatum*, *Zingiberis rhizom*a, and *Coptidis rhizome*. **(B)** Ion chromatogram of blood-entering components in BXD. **(C)** Ion chromatogram of blood-entering components in XKD (n = 6 per group).

The compositions of BXD and XKD were illustrated in Figure 1A. Comparative analysis of BXD water decoction, medicated serum, and model group serum was performed using both positive and negative ion scanning modes, and the chromatogram of the medicated serum is shown in Figure 1B, identifying nine representative blood-entry components: jatrorrhizine, epiberberine, gingerenone A, liquiritigenin, irigenin, isoliquiritigenin, fraxetin, 6-hydroxyapigenin, and apigenin. Network pharmacology analysis based on the 46 blood-entry components of BXD predicted 182 potential therapeutic targets associated with these compounds. Intersection analysis between these targets and 1,797 known targets associated with the pathological mechanisms of GIM revealed 97 common targets. Enrichment analysis of these targets identified 141 KEGG signaling pathways (*P* < 0.05), with significant enrichment in inflammation-associated pathways including TNF, Pathways in cancer, PI3K-Akt, Apoptosis, and IL-17. These findings suggested that BXD may be associated with the modulation of inflammation-related signaling pathways in GIM (Supplementary Figure S1A).

Comparative analysis of XKD water decoction, medicated serum, and model group serum revealed the positive/negative ion chromatogram of drug-containing serum through dual ion scanning modes (Figure 1C), identifying nine representative blood-entry components: coumarin, kaempferide, 6-demethoxytangeretin, nobiletin, sinensetin, quercetin-3-O-galactoside, hexahydrocurcumin, eupatilin, and formononetin. Network pharmacology analysis based on the 44 blood-entry components of XKD predicted 127 potential therapeutic targets associated with these bioactive compounds. Intersection analysis between these targets and 1,797 known targets associated with the pathological mechanisms of GIM revealed 45 common targets. Enrichment analysis of these targets identified 112 KEGG signaling pathways (*P* < 0.05), with significant enrichment in inflammation-associated pathways including Colorectal cancer, Gastric cancer, and Transcriptional misregulation in cancer. These findings suggested that XKD may be associated with the modulation of tumorigenesis-related signaling pathways in GIM (Supplementary Figure S1B).

### 3.2 Xinkai Kujiang formula ameliorated GIM lesions in rats

**FIGURE 2:**
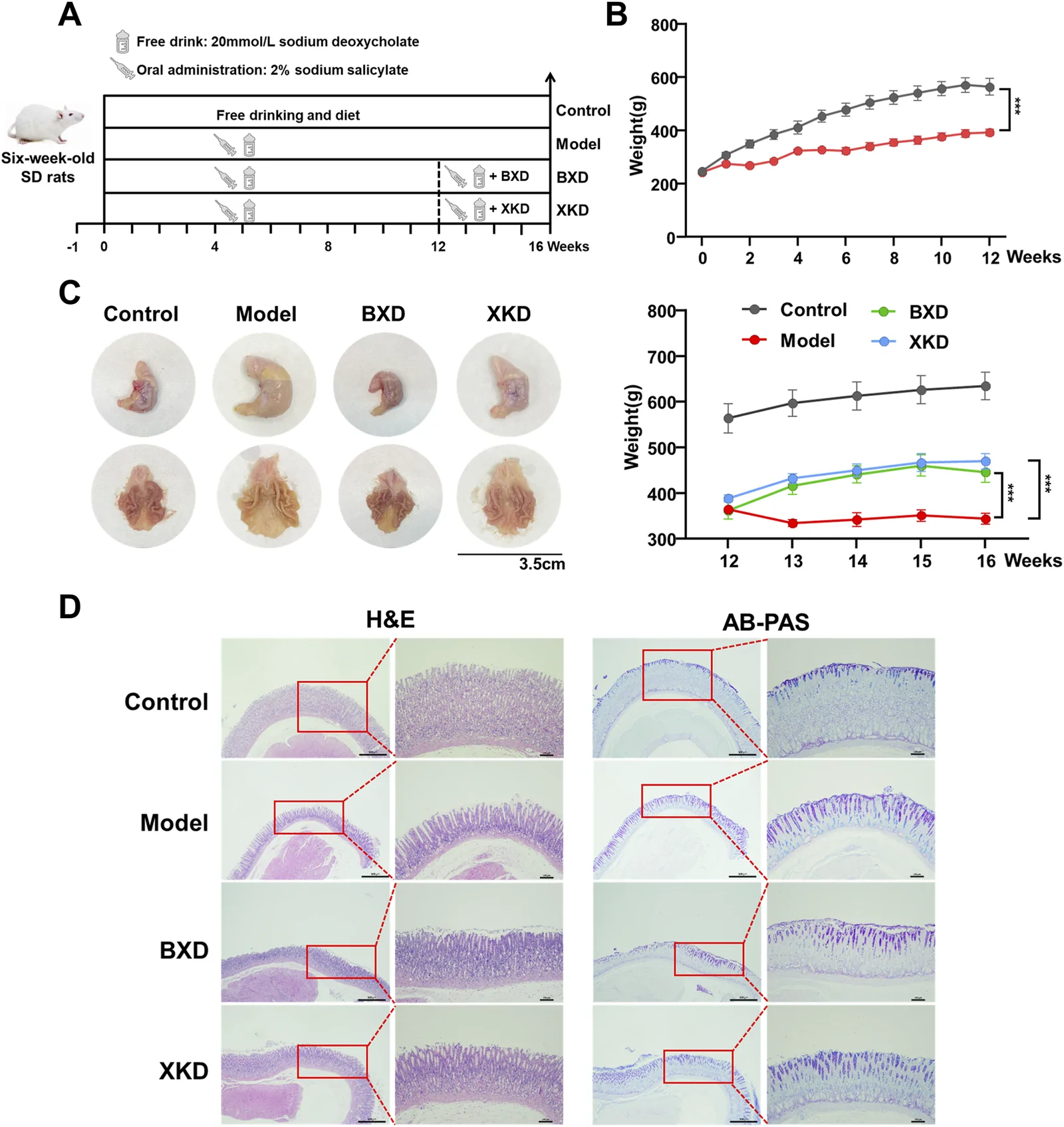
Xinkai Kujiang formula ameliorated GIM lesions in rats. **(A)** Illustration of the experimental design. **(B)** Effects of Xinkai Kujiang formula on body weight in GIM rats. **(C)** Xinkai Kujiang formula improved the morphologic lesions of the gastric mucosa. **(D)** H&E and AB-PAS staining of gastric mucosa (Magnification: ×4 , Scale bar: 500 μm; Magnification: ×10 , Scale bar: 100 μm). n = 6 per group, ^***^ *P* < 0.001.

Following 4-week intervention with the Xinkai Kujiang formula (Figure 2A), the model group exhibited significantly lower body weight compared to the control group, whereas both BXD and XKD groups showed a significant increase in body weight relative to the model group (Figure 2B). Observation of the overall gastric morphology revealed that rats in the control group had a normal corpus with smooth gastric mucosa, reddish color, and neatly arranged folds; in contrast, the model group exhibited a distended corpus with pale and thin gastric mucosa and a marked reduction in fold number. Both the BXD and XKD groups showed an improvement in gastric morphology, with restoration of smooth pink mucosa and increased fold number (Figure 2C). Histopathological analysis of gastric antral tissues demonstrated well-organized glandular architecture without infiltration, edema, or atrophy in the control group. The model group exhibited severe pathological alterations including hemorrhage and marked glandular atrophy. Importantly, interventions with BXD and XKD preserved glandular structural integrity with proper cellular alignment (Figure 2D). AB-PAS staining revealed significantly increased goblet cell-derived blue-stained secretions (indicative of intestinal metaplasia) in the model group versus controls. The intensity of blue staining in goblet cells in the gastric mucosa was significantly reduced in both the BXD and XKD groups (Figure 2D). These findings suggested that BXD and XKD, as Xinkai Kujiang formulas, effectively ameliorated gastric mucosal pathology in GIM rats.

### 3.3 Effects of Xinkai Kujiang formula on gastric mucosal microbiota in GIM rats

**FIGURE 3:**
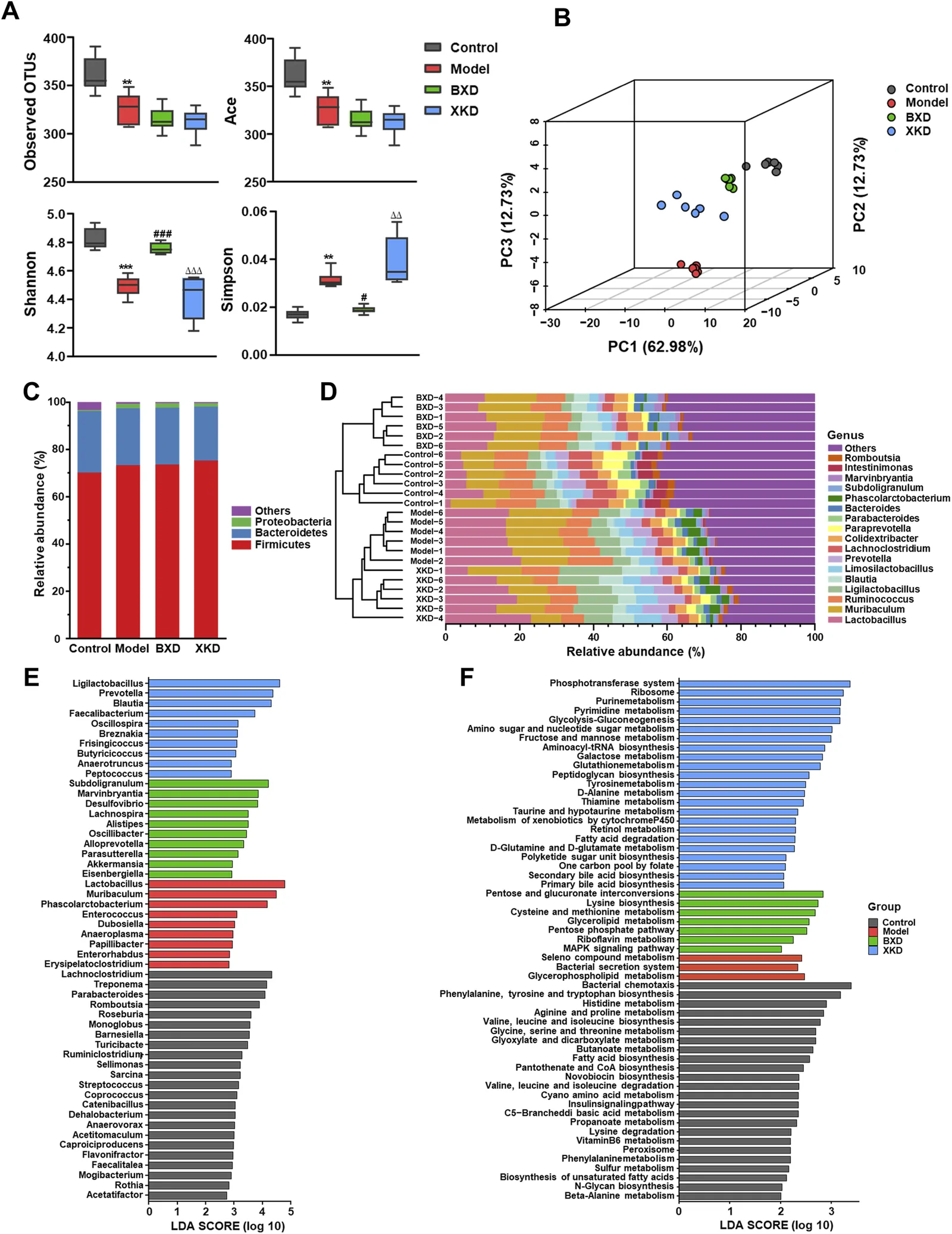
Effects of Xinkai Kujiang formula on gastric mucosal microbiota in GIM rats. **(A)** Effects of Xinkai Kujiang formula on alpha diversity of gastric mucosal bacteria in GIM rats. **(B)** Effects of Xinkai Kujiang formula on beta diversity of gastric mucosal bacteria in GIM rats. **(C)** Effects of Xinkai Kujiang formula on dominant bacterial phyla of gastric mucosa in GIM rats. **(D)** Hierarchical clustering of gastric mucosal microbiota at the genus level (Weighted-UniFrac). **(E)** LDA analysis of gastric mucosal marker genera in GIM rats treated with Xinkai Kujiang formula. **(F)** Based on the predictive function of gastric mucosal microbiota, LDA analyzed the potential signaling pathways in GIM rats treated with Xinkai Kujiang formula. n = 6 per group, compared to the control group, ^**^ *P* < 0.01, ^***^ *P* < 0.001; compared to the model group, ^*#*^ *P* < 0.05, ^*###*^ *P* < 0.001; compared to the BXD group, ^ΔΔ^ *P* < 0.01, ^ΔΔΔ^ *P* < 0.001.

Analysis of gastric mucosal microbiota showed that compared with the control group, richness (Observed OTUs, Ace) and the Shannon index were significantly decreased in the model group (*P* < 0.01), while Simpson index was significantly increased (*P* < 0.01) (Figure 3A). Principal component analysis (PCA) demonstrated clear separation of gastric mucosal microbiota composition between the model and control groups. Both BXD and XKD groups were clearly separated from the model group (Figure 3B). The dominant phyla (relative abundance >1%) were Firmicutes (73.17%), Bacteroidetes (24.24%), and Proteobacteria (1.38%) in descending order (Figure 3C). The top 10 dominant genera (relative abundance >1%) included *Lactobacillus* (12.56%), *Muribaculum* (12.31%), *Ruminococcus* (8.37%), *Ligilactobacillus* (6.01%), *Blautia* (3.7%), *Limosilactobacillus* (3.63%), *Prevotella* (3.58%), *Lachnoclostridium* (3.51%), *Colidextribacter* (3.42%) and *Paraprevotella* (1.83%). Clustering analysis using the Weighted-UniFrac algorithm on dominant genera showed that the BXD group clustered with the control group, whereas the XKD group clustered with the model group (Figure 3D). Collectively, these results demonstrated that both BXD and XKD alleviated gastric mucosal microbiota dysbiosis in GIM rats, with BXD demonstrating greater efficacy in restoring microbiota profiles closer to the control group.

LDA analysis identified 52 bacterial genera present in the model, control, BXD and XKD groups. Among these, 23 genera such as *Lachnoclostridium*, *Treponema*, *Parabacteroides*, *Romboutsia*, and *Roseburia* were enriched in the control group; nine genera including *Lactobacillus*, *Muribaculum*, *Phascolarctobacterium*, and *Enterococcus* were enriched in the model group; ten genera such as *Subdoligranulum*, *Desulfovibrio*, *Lachnospira*, *Alistipes*, *Alloprevotella*, *Parasutterella*, and *Akkermansia* were enriched in the BXD group; and ten genera such as *Ligilactobacillus*, *Prevotella*, *Blautia*, *Faecalibacterium*, *Oscillospira*, and *Butyricicoccus* were enriched in the XKD group (Figure 3E). These results indicated that Xinkai Kujiang formula treatment was associated with the enrichment of specific genera in the gastric mucosa of GIM rats.

Based on the LDA analysis of the gastric mucosal microbiota, KEGG functional prediction was performed to preliminarily explore the potential molecular mechanisms of Xinkai Kujiang formula in treating GIM in rats. The results showed that the control group was mainly enriched in signaling pathways including bacterial chemotaxis, phenylalanine, tyrosine and tryptophan biosynthesis, and histidine metabolism (Figure 3F). The model group mainly enriched seleno compound metabolism, bacterial secretion system, and glycerophospholipid metabolism. The BXD group mainly enriched pentose and glucuronate interconversions, lysine biosynthesis, and cysteine and methionine metabolism. The XKD group mainly enriched phosphotransferase system, ribosome, and purine metabolism. The results demonstrated that deoxycholic acid-induced GIM in rats was associated with the downregulation of biosynthesis and metabolism of essential amino acids, and that the treatment with Xinkai Kujiang formula was able to restore the functions of these signaling pathways.

### 3.4 Effects of Xinkai Kujiang formula on intestinal mucosal microbiota in GIM rats

**FIGURE 4:**
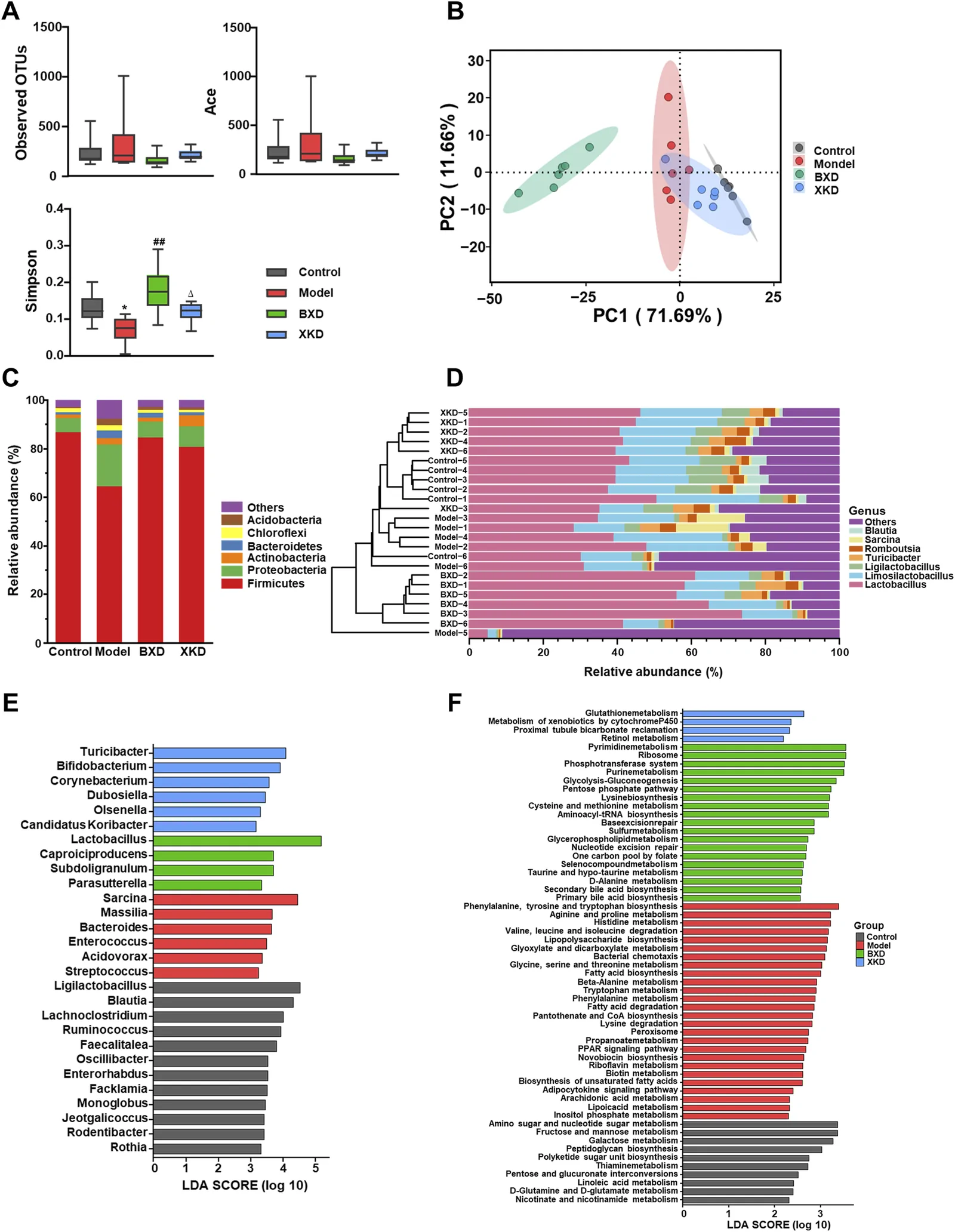
Effects of Xinkai Kujiang formula on intestinal mucosal microbiota in GIM rats. **(A)** Effects of Xinkai Kujiang formula on alpha diversity of intestinal mucosal bacteria in GIM rats. **(B)** Effects of Xinkai Kujiang formula on beta diversity of intestinal mucosal bacteria in GIM rats. **(C)** Effects of Xinkai Kujiang formula on dominant bacterial phyla of intestinal mucosa in GIM rats. **(D)** Hierarchical clustering analysis of intestinal bacterial genera was performed using the Bray-Curtis algorithm. **(E)** LDA analysis of marker intestinal genera in GIM rats treated with Xinkai Kujiang formula. **(F)** Based on the predictive function of intestinal mucosal bacteria, LDA analyzed the potential signaling pathways in GIM rats treated with Xinkai Kujiang formula. n = 6 per group. Compared with the control group, ^*^ *P* < 0.05; compared to the model group, ^##^ *P* < 0.01; compared to the BXD group, ^Δ^ *P* < 0.05.

Intestinal microbiota analysis revealed that compared with the control group, the model group displayed a significant decrease in Simpson index (*P* < 0.05), while no statistically significant changes were observed in richness indices (observed OTUs and Ace; *P* > 0.05). Following treatment with Xinkai Kujiang formula, the Simpson index was significantly elevated in the BXD group (*P* < 0.01), with a significant difference compared with the XKD group (Figure 4A, *P* < 0.05).

PCA revealed distinct separation between the model group and the control group. The XKD group exhibited an intermediate microbial composition, whereas the BXD group showed marked separation in intestinal microbiota (Figure 4B). These findings suggested that XKD ameliorated the intestinal microbiota in GIM rats, while BXD exerted distinct modulatory effects. The dominant bacterial phyla (relative abundance >1%) were ranked in descending order: Firmicutes (79.28%), Proteobacteria (9.44%), Actinobacteria (2.59%), Bacteroidetes (1.78%), Chloroflexi (1.45%), and Acidobacteria (1.43%) (Figure 4C).

Compared with the control group, the model group exhibited significantly increased Actinobacteria (*P* < 0.05), Bacteroidetes (*P* < 0.05), and Proteobacteria (*P* < 0.05) in intestinal mucosa, alongside a marked reduction in Firmicutes (*P* < 0.01). Following treatment with Xinkai Kujiang formula, the BXD group demonstrated significant decreases in Actinobacteria, Bacteroidetes, and Proteobacteria (*P* < 0.05), whereas Firmicutes abundance was notably increased (*P* < 0.05). The XKD group also showed a pronounced reduction in Bacteroidetes (*P* < 0.001). Dominant bacterial genera (relative abundance >1%) were presented in descending order: *Lactobacillus* (43.08%), *Limosilactobacillus* (17.45%), *Ligilactobacillus* (4.83%), *Turicibacter* (2.76%), *Romboutsia* (2.48%), *Sarcina* (2.02%), and *Blautia* (1.42%). Cluster analysis of the dominant genera using the Bray-Curtis algorithm showed that the XKD group clustered with the control group (Figure 4D). These results suggested that Xinkai Kujiang formula ameliorated microbiota dysbiosis in GIM rats. Moreover, XKD restored microbiota profiles closer to those of the control group, demonstrating greater efficacy than BXD.

LDA analysis identified 27 bacterial taxa distinguishing among the model, control, BXD and XKD groups. Among these, 12 genera were enriched in the control group, including *Ligilactobacillus*, *Blautia*, *Lachnoclostridium*, *Ruminococcus*, *Faecalitalea*, and *Oscillibacter*. Six genera were enriched in the model group, including *Sarcina*, *Massilia*, *Bacteroides*, *Enterococcus*, *Acidovorax*, and *Streptococcus*. Four genera showed enrichment in the BXD group, including *Lactobacillus*, *Caproiciproducens*, *Subdoligranulum*, and *Parasutterella*. Six genera were enriched in the XKD group, including *Turicibacter*, *Bifidobacterium*, *Corynebacterium*, *Dubosiella*, *Olsenella*, and *Candidatus Koribacter* (Figure 4E). These results indicated that Xinkai Kujiang formula was associated with the enrichment of specific genera in the intestinal microbiota of GIM rats.

Based on the LDA analysis of the intestinal microbiota, KEGG functional prediction was performed to explore the potential mechanisms of Xinkai Kujiang formula in treating GIM in rats. The control group was enriched in pathways including amino sugar and nucleotide sugar metabolism, fructose and mannose metabolism, and galactose metabolism (Figure 4F). The model group showed dominant enrichment in phenylalanine, tyrosine and tryptophan biosynthesis, arginine and proline metabolism, and histidine metabolism. The BXD group was mainly enriched in pyrimidine metabolism, ribosome, and phosphotransferase system pathways, while the XKD group was enriched in glutathione metabolism, metabolism of xenobiotics by cytochrome P450, and proximal tubule bicarbonate reclamation. These results suggested that deoxycholic acid-induced GIM rats exhibited upregulated biosynthesis pathways of essential amino acids and fatty acids in gastric mucosa. BXD and XKD exerted distinct therapeutic mechanisms: BXD upregulated energy metabolism and DNA repair pathways, whereas XKD enhanced biotransformation pathways.

### 3.5 Serum metabolomics analysis of the Xinkai Kujiang formula in GIM rats

**FIGURE 5:**
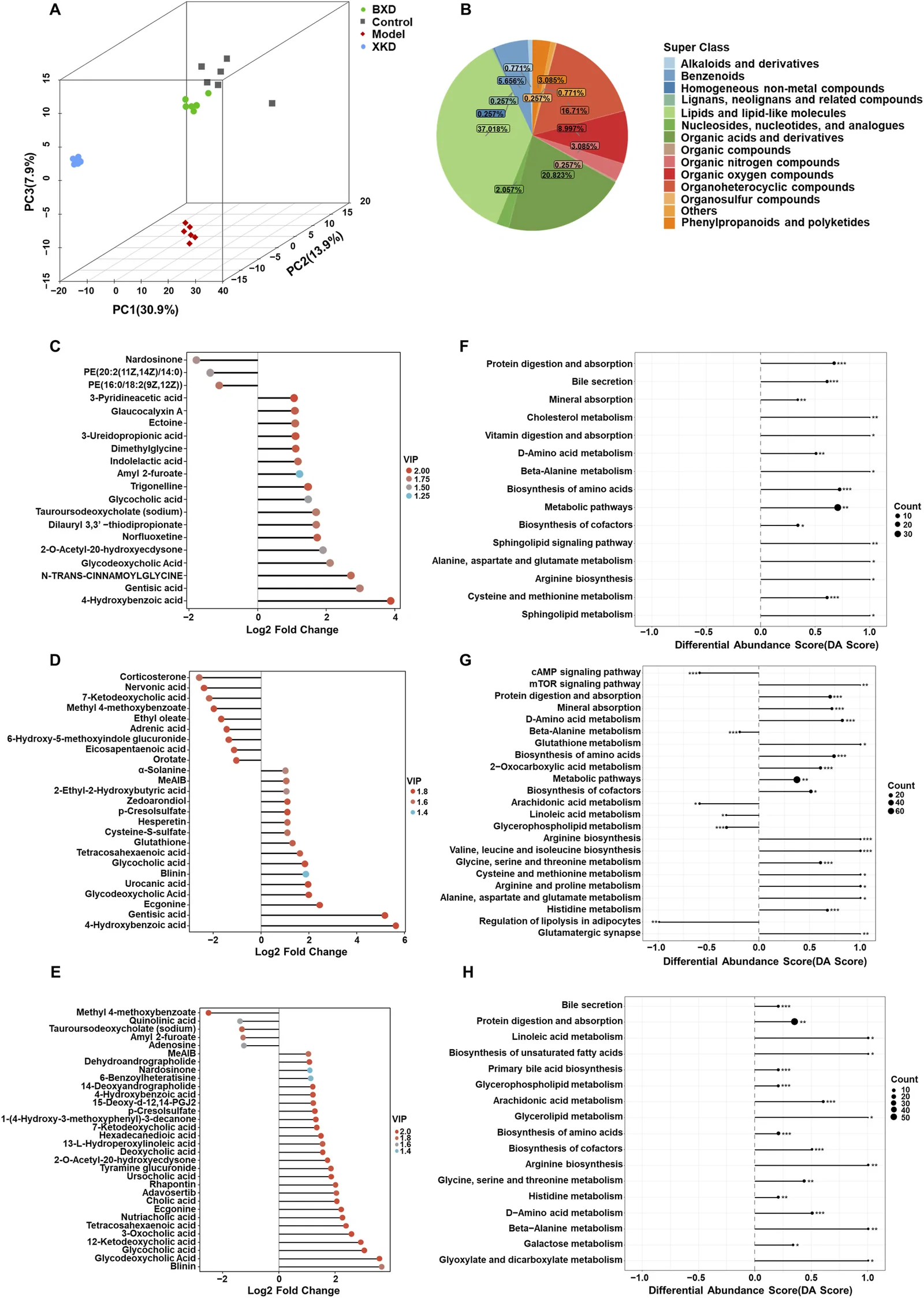
Serum metabolomics analysis of the Xinkai Kujiang formula in GIM rats. Serum untargeted metabolomics analysis was performed using LC-MS/MS. **(A)** Unsupervised PCA of serum metabolome across four groups. **(B)** Classification and composition of serum metabolites. **(C)** Serum differential metabolites of GIM rats. **(D)** Serum differential metabolites of GIM rats treated with BXD. **(E)** Serum differential metabolites of GIM rats treated with XKD. KEGG pathway enrichment analysis was performed based on differential metabolites. **(F)** Potential KEGG signaling pathway in GIM rats. **(G)** Potential KEGG signaling pathway in GIM rats treated with BXD. **(H)** Potential KEGG signaling pathway in GIM rats treated with XKD. Differential Abundance Score (DA Score) was defined as the ratio of the difference between up- and downregulated metabolites to the total annotated metabolites in a pathway, reflecting the overall change of all differential metabolites in the pathway. The length of the segment represents the absolute value of DA score, and the dot represents the number of differential metabolites in the pathway. n = 6 per group, ^*^ *P* < 0.05, ^**^ *P* < 0.01, ^***^ *P* < 0.001.

Serum untargeted metabolomics analysis based on LC-MS/MS showed 599 metabolites in four groups. PCA showed clear separation between the model and control groups. Both the BXD group and the XKD group were significantly separated from the model group, with the BXD group being closer to the control group (Figure 5A). Serum metabolite composition showed that 37.08% belonged to lipids and lipid-like molecules, 20.82% to organic acids and derivatives, 16.71% to organic heterocyclic compounds, 8.997% to organic oxygen compounds, 5.656% to benzenoids, 3.085% to phenylpropanoids and polyketides, and 3.085% to organic nitrogen compounds (Figure 5B).

Significant differential metabolites were defined as FC ≥ 2 or FC ≤ 1/2, P < 0.05 and VIP ≥1. Compared with the control group, 17 metabolites such as 4-hydroxybenzoic acid, glycodeoxycholic acid, tauroursodeoxycholic acid, glycocholic acid, three-indoleacetic acid and dimethylglycine were significantly upregulated in the model group (Figure 5C). After treatment of Xinkai Kujiang formula, 17 metabolites such as blinin, glutathione and hesperetin were significantly upregulated in the BXD group, while nine metabolites such as orotate, ethyl oleate and 7-ketodeoxycholic acid were significantly downregulated in the BXD group (Figure 5D); 27 metabolites including blinin, 3-oxalic acid and rhapontin were significantly upregulated in the XKD group, while five metabolites including adenosine, amyl 2-furoate and tauroursodeoxycholic acid were significantly downregulated in the XKD group (Figure 5E).

KEGG enrichment analysis of differential metabolites was conducted to investigate the influence of the Xinkai Kujiang formula on serum metabolite functions. Compared with the control group, 15 pathways were significantly enriched in the model group, including protein digestion and absorption, bile secretion and cholesterol metabolism (Figure 5F). Compared with the model group, 23 pathways were significantly enriched in the BXD group, including 17 pathways such as 2-oxocarboxylic acid metabolism, mTOR signaling pathway and glutathione metabolism, cAMP signaling pathway; six pathways including glycerophospholipid metabolism, linoleic acid metabolism and arachidonic acid metabolism were inhibited (Figure 5G); in the XKD group, 17 pathways were significantly enriched with their functions enhanced, including primary bile acid biosynthesis, glyoxylate and dicarboxylate metabolism, biosynthesis of unsaturated fatty acids, and galactose metabolism (Figure 5H). The results suggested that the effects of BXD and XKD on serum metabolome in GIM rats were significantly different.

### 3.6 Transcriptomic analysis of gastric mucosa in GIM rats treated with the Xinkai Kujiang formula

**FIGURE 6:**
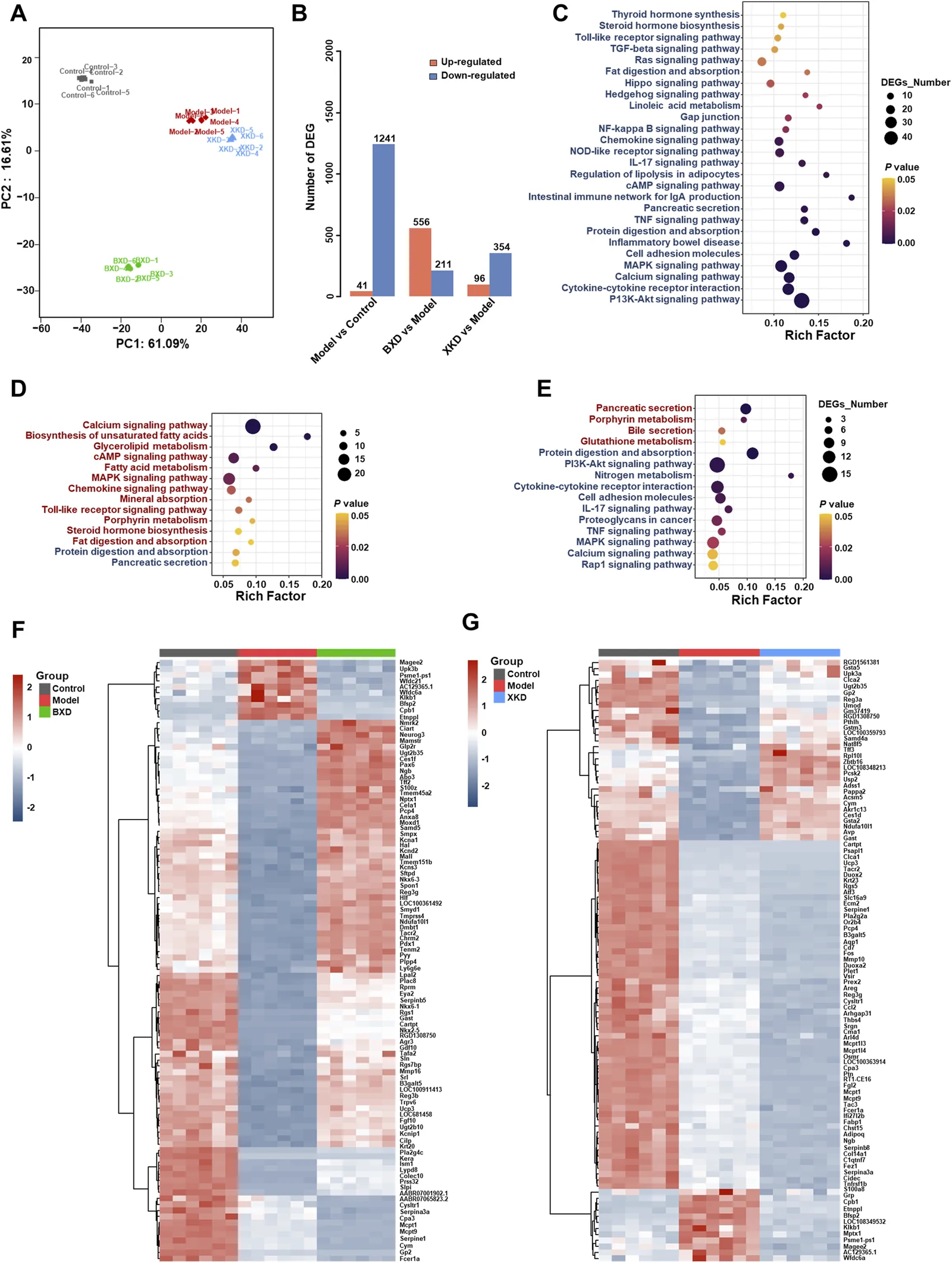
Transcriptomic analysis of gastric mucosa in GIM rats treated with the Xinkai Kujiang formula. **(A)** Xinkai Kujiang formula significantly altered the gastric mucosal transcriptome. **(B)** DEGs of gastric mucosa in GIM rats treated with Xinkai Kujiang formula. **(C)** KEGG pathway enrichment analysis of DEGs between the control and model group. **(D)** KEGG pathway enrichment analysis of DEGs between the model and BXD group. **(E)** KEGG pathway enrichment analysis of DEGs between the model and XKD group. **(F)** Hierarchical clustering of gastric mucosal DEGs in GIM rats treated with BXD. **(G)** Hierarchical clustering of gastric mucosal DEGs in GIM rats treated with XKD. Figure C–E, red represents upregulated pathways, while blue represents downregulated pathways. n = 6 per group.

Gastric mucosal transcriptome analysis showed distinct gene expression pattern between the model and control groups, and both the BXD group and the XKD group were clearly separated from the model group (Figure 6A). Compared with the control group, a total of 41 genes were upregulated and 1,241 genes were downregulated in the model group (Supplementary Table S1). Relative to the model group, 556 genes were upregulated and 211 genes were downregulated in the BXD group (Supplementary Table S2), whereas 96 genes were upregulated and 254 genes were downregulated in the XKD group (Figure 6B; Supplementary Table S3). KEGG pathway enrichment analysis revealed that, compared with the control group, all 26 enriched pathways in the model group were significantly downregulated, including calcium signaling pathway, cAMP signaling pathway, MAPK signaling pathway, chemokine signaling pathway, protein digestion and absorption, pancreatic secretion, and fat digestion and absorption (Figure 6C; Supplementary Table S4). Relative to the model group, the BXD group restored the functions of 12 signaling pathways, including calcium signaling pathway, cAMP signaling pathway, MAPK signaling pathway, chemokine signaling pathway, and fat digestion and absorption (Figure 6D; Supplementary Table S5). The XKD group enhanced the functions of four signaling pathways, including pancreatic secretion, porphyrin metabolism, bile secretion, and glutathione metabolism (Figure 6E; Supplementary Table S6). These results suggested that Xinkai Kujiang formula could restore the gastric mucosal transcriptome in GIM rats, with the BXD group showing stronger effects than the XKD group, which is consistent with the findings from the gastric mucosal microbiota analysis.

Hierarchical clustering analysis displayed the top 100 DEGs. Based on the analysis of the control, model, and BXD groups, the expression of trefoil factor 2 (*Tff2*), surfactant protein D (*Sftpd*), deleted in malignant brain tumors 1 (*Dmbt1*), secretory leukocyte peptidase inhibitor (*Slpi*), and regenerating family members 3 beta/gamma (3β/3γ) was significantly reduced in the gastric mucosa of the model group. The BXD group markedly reversed the expression levels of these genes (Figure 6F). Comparing the control, model, and XKD groups, the expression of trefoil factor 3 (*Tff3*), regenerating islet-derived 3 alpha (*Reg3a*), zinc finger and BTB domain containing 16 (*Zbtb16*), and arginine vasopressin (*Avp*) was significantly downregulated in the model group, whereas S100 calcium binding protein A8 (*S100a8*) was significantly upregulated. After the XKD treatment, the expression of *Tff3*, *Reg3a*, *Zbtb16*, and *Avp* was further upregulated, while *S100a8* was markedly downregulated (Figure 6G). The results suggested that the molecular mechanisms of BXD and XKD in treating GIM were markedly different. XKD may improve GIM through regulation of the gut microbiota and its metabolites.

Spearman correlation analysis was performed to explore the potential associations among gastric microbiota, intestinal microbiota, serum metabolites, and gastric mucosal DEGs following BXD and XKD treatment. In the BXD group, the gastric genus *Ruminiclostridium* was positively correlated with *Reg3b* expression (R = 0.816) and *Slpi* expression (R = 0.865) (Supplementary Figure S2A; Supplementary Table S7); additionally, intestinal genus *Lachnoclostridium* showed a positive correlation with the serum metabolite rdosinone (R = 0.701), which was further positively associated with *Reg3b* expression (R = 0.715) and *Slpi* expression (R = 0.738) (Supplementary Figure S2B; Supplementary Table S8). In the XKD group, the gastric genus *Treponema* was positively correlated with *Reg3a* expression (R = 0.804) (Supplementary Figure S2C; Supplementary Table S9), and the intestinal genus *Blautia* was positively correlated with the serum metabolite dilauryl 3,3′-thiodipropionate (R = 0.932), which was further positively associated with *Reg3a* expression (R = 0.905) (Supplementary Figure S2D; Supplementary Table S10). These findings suggest potential microbiota-metabolite-gene association patterns related to BXD and XKD treatment.

Immunohistochemical staining was used to validate selected gastric mucosal transcriptomic genes, focusing on innate immunity-related proteins, such as Bves, Dmbt1, ZO-1, and IgA. Compared with the control group, the expression of Bves, Dmbt1, and ZO-1 was decreased, whereas IgA expression was increased in the gastric mucosa of model rats. Treatment with BXD and XKD reversed these changes (Supplementary Figure S3). The results suggested that Xinkai Kujiang formula could improve the innate immune barrier function of the gastric mucosa in GIM rats, with BXD showing greater efficacy than XKD.

### 3.7 Cellular landscape analysis of gastric antral mucosa in GIM rats treated with the Xinkai Kujiang formula

**FIGURE 7:**
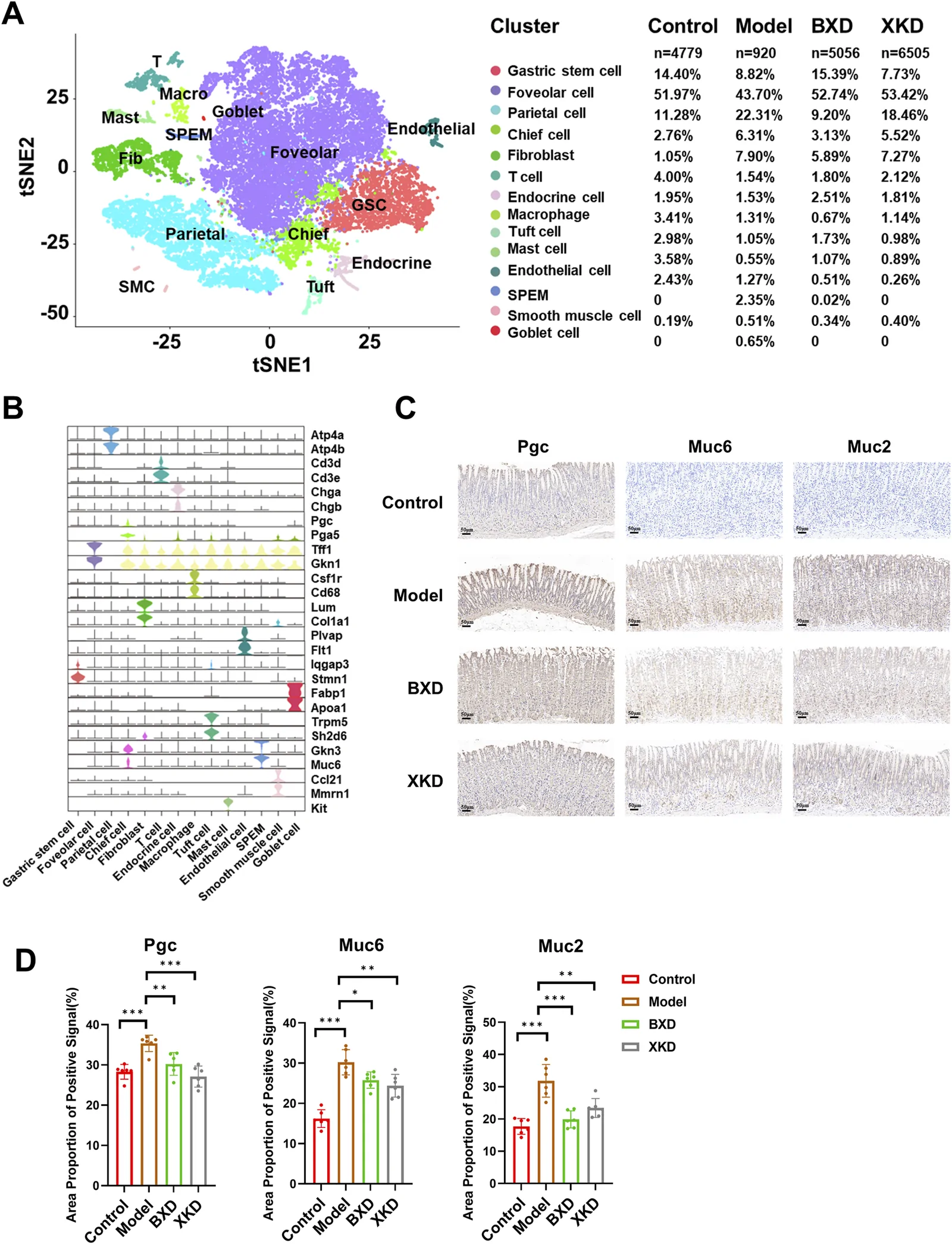
Cellular landscape analysis of gastric antral mucosa in GIM rats treated with the Xinkai Kujiang formula. **(A)** t-SNE analysis showing 14 clusters. **(B)** Marker gene analysis for the 14 clusters. **(C)** Immunohistochemical staining showing the expression of Pgc, Muc2, and Muc6 **(D)** Quantitative analysis of immunohistochemical staining, expressed as the percentage of positive area (Area%, n = 6). Magnification: ×20 , Scale bar: 50 μm, *P < 0.05, **P < 0.01, ***P < 0.001.

ScRNA-seq identified 4,779 cells in the control group, 9,203 cells in the model group, 5,056 cells in the BXD group, and 6,506 cells in the XKD group. All captured cells were partitioned into 14 clusters (Figure 7A). Fourteen cell types were defined by marker genes (Figure 7B; Supplementary Table S11), including Gastric stem cells (GSCs; *Stmn1, Iqgap3*), Foveolar cells (*Tff1, Gkn1*), Parietal cells (*Atp4a*, *Atp4b*), Chief cells (*Pgc*, *Pga5*), Fibroblasts (*Lum*, *Col1a1*), T cells (*Cd3d*, *Cd3e*), Endocrine cells (*Chga, Chgb*), Macrophages (*Cd68*, *Csf1r*), Tuft cells (*Trpm5, Sh2d6*), Mast cells (*Kit*), Endothelial cells (*Flt1*, *Plvap*), Spasmolytic polypeptide-expressing metaplasia cells (SPEM; *Gkn3, Muc6*), Smooth muscle cells (SMC; *Ccl21, Mmrn1*), and Goblet cells (*Fabp1, Apoa1*). Among these, SPEM cells were present in the model and BXD groups, whereas goblet cells appeared exclusively in the model group. The presence of these two cell types indicated abnormal intestinal metaplasia characterized by the replacement of gastric epithelium with intestinal epithelial mucosa within the stomach. GSCs were enriched in the control and BXD groups but reduced in the model and XKD groups. Foveolar cells were enriched in the control, BXD, and XKD groups, while reduced in the model group. Parietal cells were more abundant in the model and XKD groups but less represented in the control and BXD groups. Fibroblasts showed lower abundance in the control group but were enriched in the model, BXD, and XKD groups. T cells and macrophages were enriched in the control group (Figure 7A).

Immunohistochemical staining revealed that, compared with the control group, the expression of Pepsinogen C (a marker protein of chief cells, Pgc), Mucin 6 (Muc6), and Mucin 2 (metaplasia-related proteins, Muc2) was increased in the model group. Following BXD and XKD treatment, their expression levels were markedly decreased (Figures 7C, D). Upregulation of Muc6 and Muc2 marks the progression of SPEM and intestinal metaplasia, respectively, consistent with the appearance of goblet cells. Downregulation of Muc6 and Muc2 indicated that the Xinkai Kujiang formula blocked the transformation of SPEM toward intestinal metaplasia. Collectively, these results suggested that the Xinkai Kujiang formula normalized the cellular composition of the antral mucosa in GIM rats by reshaping the gastric mucosal cellular landscape, inhibiting abnormal differentiation of SPEM and goblet cells, thereby rendering the cellular profile close to that of the normal control group.

Cell cycle comprises interphase (divided into G1, S, and G2 phases) and mitosis (M phase), and its proper regulation is essential for cell growth, development, and tissue homeostasis. Cell cycle analysis of the antral mucosa showed 56.8% of cells in G1, 22.0% in S, and 21.2% in G2/M phase (Supplementary Figure S4A; Supplementary Table S12). Stratified cell cycle analysis revealed GSCs were predominantly distributed in G2/M phase, indicating a critical role in mucosal self-renewal and injury repair. In the BXD group, endothelial cells exhibited lower G1 proportion (42.3% vs. ∼70% in other groups) and higher G2/M proportion (27%); notably, no endothelial cells in the XKD group were detected in G2/M phase. Parietal cells showed similar G1 proportions in the control and BXD group (43.6% and 37.2%, respectively), while model and XKD groups displayed higher G1 fractions (56% and 53.4%, respectively), accompanied by corresponding changes in G2/M phase. Additionally, model group had a lower G1 proportion (52.0%) than other groups (63.6%–75.0%) (Supplementary Figure S4B), suggesting BXD treatment may modulate the cell cycle distribution of parietal cells in GIM rats. Bockerstett et al. (2020) proposed that SPEM represents a regenerative gastric mucosal lesion that may precede intestinal metaplasia or gastric adenocarcinoma under chronic inflammatory conditions. Differential gene expression analysis identified significantly upregulated or downregulated genes in SPEM cells across different phases. Specifically, G2/M-phase SPEM cells exhibited upregulated Zfp106, Gkn1, Gkn2, and Tff1, and downregulated Igf2r, Tmco3, Gnpnat1, and Eef2 (Supplementary Figure S4C), providing new insights into SPEM development mechanisms.

### 3.8 Xinkai Kujiang formula alleviating GIM linked with the HIF-1α/VEGF pathway

**FIGURE 8:**
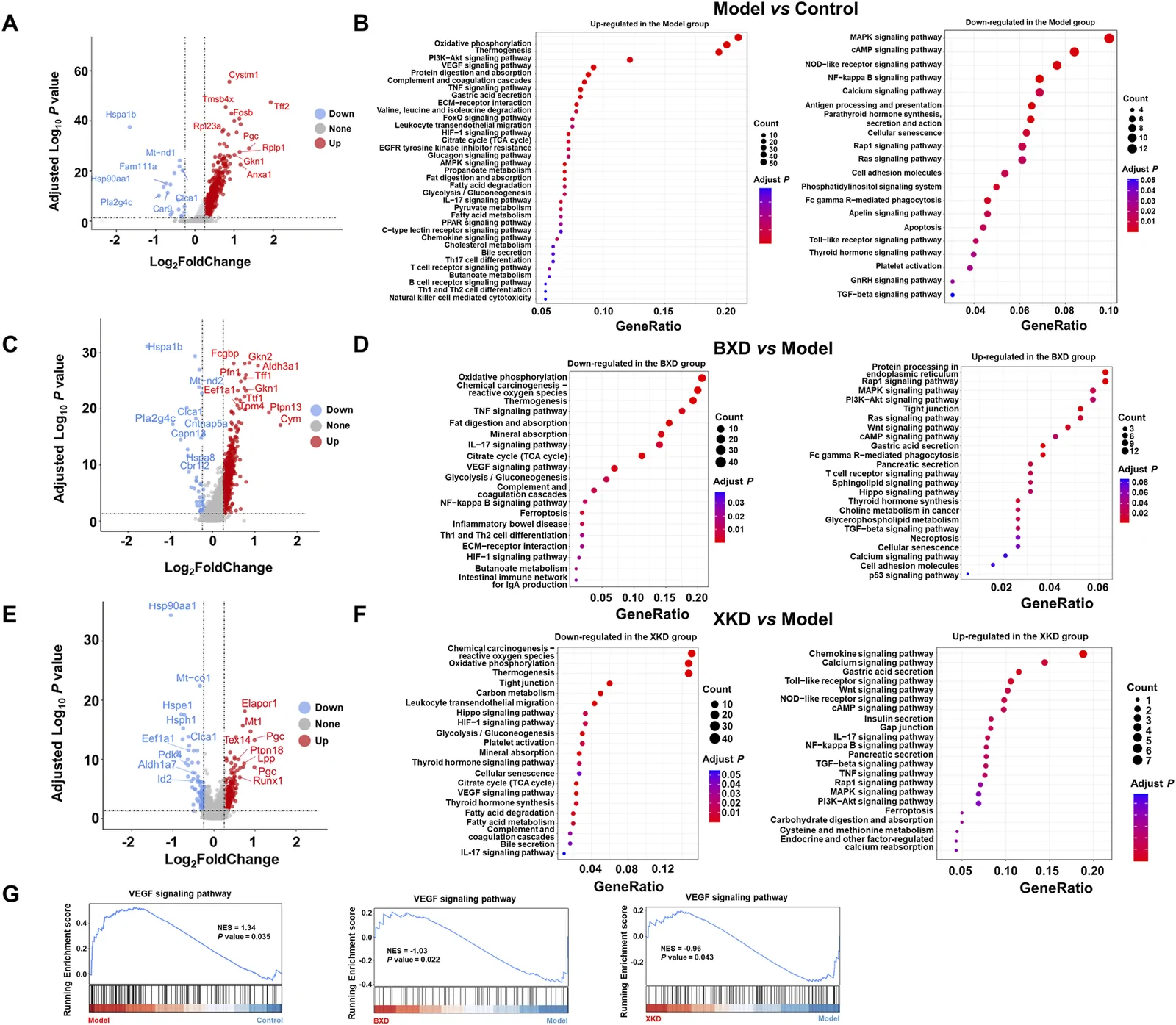
Xinkai Kujiang formula alleviating GIM linked with the HIF-1α/VEGF pathway. **(A)** Volcano plot of differential gene expression between the control and model groups. **(B)** KEGG analysis of upregulated and downregulated genes between the control and model groups. **(C)** Volcano plot of differential gene expression between the model and BXD groups. **(D)** KEGG analysis of upregulated and downregulated genes between the model and BXD groups. **(E)** Volcano plot of differential gene expression between the model and XKD groups. **(F)** KEGG analysis of upregulated and downregulated genes between the model and XKD groups. **(G)** GSEA indicated significant enrichment of the VEGF signaling pathway in the model group.

Differential gene expression analysis showed 508 significantly upregulated and 181 significantly downregulated genes in the model group compared with the control group (Figure 8A). KEGG pathway enrichment analysis was performed separately for these up- and downregulated genes (Figure 8B). Correspondingly, 345 genes were upregulated and 281 were downregulated in the BXD group compared with the model group (Figure 8C), while 76 genes were upregulated and 423 were downregulated in the XKD group (Figure 8E); KEGG pathway enrichment analyses were presented in (Figure 8D; Figure 8F). Among the signaling pathways that were upregulated in the model group, treatment with BXD and XKD resulted in significant downregulation, including oxidative phosphorylation, thermogenesis, HIF-1 signaling pathway, VEGF signaling pathway, and IL-17 signaling pathway. These findings suggested that Xinkai Kujiang formula may alleviate GIM by inhibiting energy metabolism and angiogenesis. Conversely, BXD and XKD upregulated the pathways that were downregulated in the model group (Rap1, MAPK, cAMP, and NF-κB signaling pathways), indicating a potential mechanism for improving GIM via enhanced cell-cell adhesion.

Previous studies have indicated that neovascularization exacerbates gastric mucosal inflammation and damage, with the VEGF and HIF-1 signaling pathways playing key regulatory roles (Hicklin and Ellis, 2005). Gene Set Enrichment Analysis (GSEA) showed the VEGF pathway was upregulated in the model group but downregulated in BXD and XKD groups (Figure 8G). Thus, here boldly speculated that the Xinkai Kujiang formula may alleviate GIM by inhibiting VEGF and HIF-1 signaling pathways, thereby reducing neovascularization, inflammatory cell infiltration, and energy metabolism.

CellChat network analysis revealed that in the control group, endothelial cells primarily served as recipient cells, receiving signals from fibroblasts, parietal cells, chief cells, and endocrine cells (Supplementary Figure S5A). In the model group, cell-cell communication was more complex, with goblet cells, macrophages, fibroblasts, endothelial cells, GSCs, and endocrine cells functioning as both ligand-sending and signal-receiving cells (Supplementary Figure S5B). After Xinkai Kujiang formula treatment, the BXD group exhibited intercellular communication patterns similar to the control group (Supplementary Figure S5C), whereas the XKD group exhibited markedly more fibroblast-to-macrophage and macrophage-to-endothelial cell communications than other cell types (Supplementary Figure S5D). Further analysis via the netVisual_bubble function showed that the model group had a significantly higher probability of VEGF signaling-related ligand-receptor communications than the other three groups (Supplementary Figure S5E). Several genes involved in VEGF pathway were notably altered, the upregulated genes (*Vegf*, *Mapk12*, *Src*, and *Akt1*) in the model group were markedly reversed in both BXD and XKD groups (Supplementary Figure S5F). To further validate the hyperactivation of VEGF signaling and its downstream pro-angiogenic and hypoxic responses during GIM progression, immunohistochemical staining showed that significantly elevated protein expression levels of VEGF, HIF-1α, and CD31 were observed in the model group, both BXD and XKD treatment effectively reduced the expression levels of VEGF, HIF-1α, and CD31 (Supplementary Figure S5G; Supplementary Figure S5H). Collectively, these results indicated that the therapeutic efficacy of the Xinkai Kujiang formula against GIM is closely associated with the blockade of the VEGF/HIF-1α angiogenic signaling axis, which ameliorates abnormal vascular remodeling and disordered intercellular communication in the gastric mucosal microenvironment.

## 4 Discussion

Numerous clinical studies have shown the clinical efficacy of TCM in preventing and treating PLGC, a meta-analysis of 12 randomized controlled trials (1,140 CAG patients) showed TCM significantly improved clinical efficacy in CAG (Weng et al., 2023). The larger meta-analysis of 26 trials (1,985 PLGC patients) demonstrated that BXD could significantly alleviate the clinical symptoms, such as gastric distension and belching (Cao et al., 2020), but the detailed mechanisms remain challenging to elucidate. The present study utilized multi-omics technologies (microbiome, metabolomics, transcriptomics, and scRNA-seq) to investigate the potential molecular and cellular changes associated with BXD and XKD treatment in GIM rats. Microbiome analysis showed that gastric mucosal microbiota richness and diversity were significantly reduced in GIM rats; BXD treatment resulted in gastric mucosal microbiota structure more similar to that of the control group than XKD treatment. In contrast, intestinal microbiota diversity was significantly reduced in GIM rats, and XKD treatment showed a greater effect in restoring intestinal microbiota composition toward that of the control group. These results suggest that BXD and XKD may be associated with differential modulation of gastric and intestinal microbiota, respectively.

BXD treatment reduced the abundance of gastric *Lactobacillus* and *Enterococcus* in GIM rats, while increasing the abundance of short-chain fatty acid-producing bacteria (*Akkermansia*, *Alistipes*, *Lachnospira*). XKD treatment was associated with enrichment of intestinal bacteria (*Olsenella*, *Dubosiella*, *Corynebacterium*, *Bifidobacterium*, *Turicibacter*). Previous studies have shown that *Lactobacillus* accounts for ∼25% of normal gastric mucosal microbiota (Li et al., 2021b) but its relative abundance reaches 35.2%–97.0% in Hp-negative GC patients (Gantuya et al., 2020), and gradually increases in patients with chronic gastritis, GIM, and GC (Vinasco et al., 2019). Animal experiments have also found significantly increased gastric *Lactobacillus* abundance in GIM rats with bile reflux (Jin et al., 2022), suggesting that BXD inhibited the gastric *Lactobacillus* and improved gastric precancerous lesions.

In addition, the acetic acid-producing bacteria *Alistipes* exerted protective effects in colitis, depression, liver fibrosis, and cardiovascular disease, and could even enhance the efficacy of malignant tumor immunotherapy (Parker et al., 2020). Increased abundance of the butyrate-producing bacteria *Lachnospira* (Yu et al., 2024) and *Akkermansia* was linked with the improvement of gastrointestinal mucosal lesions (Cani et al., 2022). These findings provided potential marker bacteria for clarifying the biological mechanisms of the Xinkai Kujiang formula in preventing and treating gastrointestinal lesions.

Recent studies showed that the gastrointestinal microbiota regulates host cell differentiation and gene expression via metabolite-immune axes and microbe-host gene interactions, which played a critical role in the gastric mucosal lesion pathogenesis. For example, deoxycholic acid (DCA)-mediated dysbiosis could activate STAT3 signaling pathway, disrupt gastric bile acid metabolism, and drive aberrant expression of intestinal metaplasia-related transcription factors (Jin et al., 2022). ScRNA-seq further revealed that *H. pylori* (Hp)-positive gastric mucosal microenvironments enhanced the intercellular communication and enriched intestinal mucosa marker genes (e.g., *FABP1*, *DMBT1*), suggesting microbial infection promotes gastric mucosal metaplastic transformation by reprogramming the host transcriptional network (Li et al., 2024). Further study showed that *Segmented filamentous bacteria* (SFB) activated the STAT3 signaling pathway in dendritic cells, inducing Th17 cell differentiation and IL-22 secretion to stimulate intestinal crypt stem cell proliferation. In contrast, *Clostridia* promoted regulatory T cell (Treg) differentiation, suppressed excessive inflammation to maintain mucosal homeostasis (Ivanov et al., 2009).

Gastric mucosal transcriptome and cellular atlas analysis revealed significant intergroup differences in cell composition. SPEM and goblet cells were identified in the model group, consistent with previous studies on GIM-associated gastric mucosal alterations (Goldenring, 2023). These cells were considered as regenerative gastric mucosal lesions and potential precursors of intestinal metaplasia or gastric adenocarcinoma under chronic inflammation, indicating a trend toward disease progression. *MUC6* was a characteristic SPEM marker (Li et al., 2021b). *MUC2* was predominantly expressed in colonic goblet cells and absent from normal gastric mucosa, and its expression in gastric mucosa was closely associated with GC development (Yang et al., 2022). Both XKD and BXD treatment markedly reduced MUC6 and MUC2 expression and reversed their aberrant expression patterns, and BXD could even restore the gastric antral mucosal cell profile of GIM rats close to that of the control group and ameliorate pathological alterations.

Signaling pathway enrichment analysis showed that both BXD and XKD treatment could upregulate three pathways (calcium, cAMP, and MAPK signaling pathways) and downregulate seven pathways (chemical carcinogenesis–reactive oxygen species, oxidative phosphorylation, thermogenesis, VEGF, HIF-1, IL-17, and complement and coagulation cascades). The cAMP pathway, a classic route that regulated downstream pathways via intracellular cAMP levels, could protect the gastric mucosal barrier by enhancing intercellular communication, reducing immune activation and pro-inflammatory cytokine production, and increasing the expression of cytoprotective proteins (e.g., HSP70) (Moawad et al., 2019). In this study, cAMP pathway was downregulated in the model group but significantly upregulated after Xinkai Kujiang formula treatment. *Bves* interacted with cAMP and ZO-1 to maintain epithelial integrity (Han et al., 2019), and clinical study found that *Popdc1* (also named as *Bves*) expression was decreased in GIM and GC tissues, negatively correlating with *LGR5*, *OLFM4*, and *CDX2* (Gingold-Belfer et al., 2021). Thus, Xinkai Kujiang Formula alleviated GIM at least partially by restoring cAMP/Popdc1 signaling pathway and stabilizing epithelial barrier function.

Furthermore, here found that Xinkai Kujiang formula significantly upregulated genes related to gastric mucosal innate immunity in GIM rats, including *Tff2*, *Dmbt1*, *Reg3b*, and *Reg3g*. *Dmbt1* mediated the intestinal epithelial cell resisting *Salmonella enterica* invasion (Rosenstiel et al., 2007), while *Reg3b* and *Reg3g* could directly target Gram-positive bacteria (Udomsopagit et al., 2020). Zhuang et al. (2022) found that ELFN1-AS1 recruits DNA methyltransferases to the Zbtb16 promoter, inducing silencing and activating the PI3K/AKT pathway to promote GC malignant progression. Overexpression of *Reg3a* inhibits PI3K/AKT and GSK3β pathways, suppressing GC proliferation (Qiu et al., 2018), and was positively correlated with *Dmbt1* in GC patients, modulating cell proliferation via *Dmbt1* (Wang et al., 2021). These data suggested that Xinkai Kujiang formula could restore gastric mucosal barrier integrity and inhibit tumor-related genes.

Disordered mucosal innate immunity and compromised epithelial barrier function constitute the core pathological drivers of GIM (Asan et al., 2016). Meanwhile, sustained hypoxia-triggered aberrant angiogenesis exacerbates mucosal inflammatory injury, a process tightly orchestrated by the HIF-1α/VEGF signaling axis (Yin et al., 2021). VEGF could specifically affect the proliferation and migration of vascular endothelial cells, and participate in angiogenesis and exacerbate gastric mucosal inflammation and damage (Jia et al., 2024). Dysregulated angiogenesis was a central hallmark of chronic inflammatory diseases (Carmeliet, 2005), and VEGF-mediating aberrant neovascularization was a critical mechanism for amplifying mucosal inflammation and maintaining chronic inflammation in inflammatory bowel disease (Danese et al., 2006). HIF-1α could directly regulate VEGF gene expression during tumor angiogenesis, HIF-1α upregulation promoted VEGF and COX-2 expression and CAG progression in a CAG rat model (Yin et al., 2021). In this study, both VEGF and HIF-1α were significantly downregulated by BXD and XKD treatment, which indicated that Xinkai Kujiang formula may alleviate GIM in rats by inhibiting the VEGF/HIF-1α signaling pathways to reduce neovascularization and attenuate inflammatory responses.

In summary, this study confirmed the therapeutic efficacy of Xinkai Kujiang formula in alleviating GIM and provided preliminary insights into its mechanisms via multi-omics approaches. Of course, the limitations should be acknowledged, solid functional experiments should be supplemented to verify the mechanism of action of the Xinkai Kujiang formula; the scRNA-seq should be repeated more times in each group. In future study, we will validate the major active monomers to clarify the key pharmacologically active substances underlying the efficacy of the compound.

## 5 Conclusion

The multi-omics approaches presented inconsistent molecular mechanisms underlying the therapeutic effect of BXD and XKD in spite of their similar therapeutic effect on GIM. The data provide novel mechanistic evidence and promising therapeutic targets for the clinical application of Xinkai Kujiang formula intervention of GIM.

## Data Availability

These raw data have been deposited in the Genome Sequence Archive in National Genomics Data Center, China National Center for Bioinformation / Beijing Institute of Genomics, Chinese Academy of Sciences (GSA: CRA027040) that are publicly accessible at https://ngdc.cncb.ac.cn/gsa.

## Supplementary material

SUPPLEMENTARY FIGURE S1 Network pharmacology analysis of blood-entry components in BXD and XKD. **(A)** Network pharmacology analysis of blood-entering components in BXD. **(B) **Network pharmacology analysis of blood-entering components in XKD.

SUPPLEMENTARY FIGURE S2 BXD and XKD treatment shaped different networks of gastrointestinal microbiota, serum metabolites and gastric DEGs. Based on the different bacterial genera, serum metabolites and gastric DEGs, Spearman correlation analysis was conducted to build the network with |R| > 0.7 and P < 0.05. **(A)** The network between gastric microbiota and DEGs in BXD group. **(B)** The network among gut microbiota and DEGs in BXD group. **(C)** The network between gastric microbiota and DEGs in XKD group. **(D)** The network among gut microbiota and DEGs in XKD group. In the network, the red nodes were bacterial genera, the purple nodes were serum metabolite, and the blue nodes were gastric DEGs. The red lines were significantly positive correlations, and the blue lines were significantly negative correlations.

SUPPLEMENTARY FIGURE S3 Key gene validation in GIM rats. **(A)** Immunohistochemical staining of Bves, Dmbt1, ZO-1 and lgA. **(B)** Quantitative analysis of immunohistochemical staining, expressed as the percentage of positive area (Area%, n = 6). Magnification: 20 ×, Scale bar: 100 μm, *P < 0.05, **P < 0.01, ***P < 0.001.

SUPPLEMENTARY FIGURE S4 Cell cycle distribution of rat gastric antral mucosal. **(A)** t-SNE analysis showing the cell cycle distribution of 14 clusters. **(B)** Stacked barplot illustrating the cell cycle distribution of the 14 clusters across the four groups. **(C)** Differentially expressed genes that are significantly upregulated or downregulated in SPEM at different phases.

SUPPLEMENTARY FIGURE S5 Cell-cell communication and VEGF signaling pathway analysis in rats. **(A)** Interaction network of 12 clusters in the control group. **(B)** Interaction network of 14 clusters in the model group. **(C)** Interaction network of 13 clusters in the BXD group. **(D)** Interaction network of 12 clusters in the XKD group. In Figure **(A–D)**, The arrows represent ligands, pointing to receptors, and line thickness indicates the extent of communication. **(E)** Bubble plot of ligand-receptor related to angiogenesis signaling pathways. **(F)** Heatmap of angiogenesis signaling pathway related genes. **(G)** Immunohistochemical staining of VEGF, HIF-1α, and CD31. **(H)** Quantitative analysis of immunohistochemical staining, expressed as the percentage of positive area (Area%, n = 6). Magnification: 20 ×, Scale bar: 50 μm, *P < 0.05, **P < 0.01, ***P < 0.001.
